# Provider costs for prevention and treatment of cardiovascular and related conditions in low- and middle-income countries: a systematic review

**DOI:** 10.1186/s12889-015-2538-z

**Published:** 2015-11-26

**Authors:** Elizabeth D. Brouwer, David Watkins, Zachary Olson, Jane Goett, Rachel Nugent, Carol Levin

**Affiliations:** Disease Control Priorities Network, Department of Global Health, University of Washington, 325 Ninth Avenue, Box 259931, Seattle, WA 98104 USA; Department of Medicine, University of Washington, 325 Ninth Ave, Box 359780, Seattle, WA 98104 USA; School of Public Health, University of California Berkeley, 50 University Hall, #7360, Berkeley, CA 94720-7360 USA; PATH, 2201 Westlake Ave #200, Seattle, WA 98121 USA

**Keywords:** Cardiovascular disease, CVD, Systematic review, Economic evaluation, Non-communicable disease, Cost analysis

## Abstract

**Background:**

The burden of cardiovascular disease (CVD) and CVD risk conditions is rapidly increasing in low- and middle-income countries, where health systems are generally ill-equipped to manage chronic disease. Policy makers need an understanding of the magnitude and drivers of the costs of cardiovascular disease related conditions to make decisions on how to allocate limited health resources.

**Methods:**

We undertook a systematic review of the published literature on provider-incurred costs of treatment for cardiovascular diseases and risk conditions in low- and middle-income countries. Total costs of treatment were inflated to 2012 US dollars for comparability across geographic settings and time periods.

**Results:**

This systematic review identified 60 articles and 143 unit costs for the following conditions: ischemic heart disease, non-ischemic heart diseases, stroke, heart failure, hypertension, diabetes, and chronic kidney disease. Cost data were most readily available in middle-income countries, especially China, India, Brazil, and South Africa. The most common conditions with cost studies were acute ischemic heart disease, type 2 diabetes mellitus, stroke, and hypertension.

**Conclusions:**

Emerging economies are currently providing a base of cost evidence for NCD treatment that may prove useful to policy-makers in low-income countries. Initial steps to publicly finance disease interventions should take account of costs. The gaps and limitations in the current literature include a lack of standardized reporting as well as sparse evidence from low-income countries.

**Electronic supplementary material:**

The online version of this article (doi:10.1186/s12889-015-2538-z) contains supplementary material, which is available to authorized users.

## Background

Cardiovascular disease (CVD) and CVD risk conditions, such as hypertension, type 2 diabetes mellitus (T2DM), and chronic kidney disease (CKD), are the leading cause of global morbidity and mortality [1]. Increasingly larger proportions of the global population are exposed to risk of non-communicable disease (NCD), such as urbanization, tobacco use, and sedentary behavior [2]. The growing prevalence of these conditions is particularly troublesome in low- and middle-income countries (LMICs), where age-specific rates for NCD mortality are nearly twice as high as in high-income countries [3], and treatment can be very costly [4]. There is evidence that health systems in these settings are already strained by the dual burden of infectious and chronic disease, especially in resource-limited settings [5]. Many people suffering from CVD in LMICs remain untreated, or their conditions are poorly managed, due to lack of access to primary health care and high costs [6].

Efforts to improve prevention and management of NCDs in LMICs are increasing. Mortality reduction targets, such as the World Heart Federation’s 25 × 25 and the Lancet’s 40 × 30, are unprecedented in putting NCDs on the international agenda [7, 8]. The resources required in order to meet these targets in any given LMIC are less clear. Economic evaluations are especially important for settings that do not yet have policies or the infrastructure in place to address these emerging issues [9]. Unfortunately, financial and economic cost estimates of these interventions in LMICs are limited. In order to raise awareness of the economic costs of CVD and to support increased allocation of resources for addressing CVD and risk conditions, accurate cost data are needed by donors, ministries of health and economic modelers.

Recent reviews of household burden or cost-effectiveness of CVD interventions in LMICs have been published [6, 10–13]; however, there are no recent reviews, to our knowledge, of provider-incurred costs of interventions for CVD and risk conditions. We therefore reviewed literature on the provider-incurred costs for the treatment of cardiovascular and related risk conditions in health system settings of LMICs. Specifically, we consider diabetes, chronic kidney disease, hypertension and hyperlipidemia, stroke, ischemic heart disease, and non-ischemic heart diseases. We chose these conditions because of their interrelated risk factors and the large overlap in clinical resources that are required to management them. We will refer to the entire grouping as “CVRD” (cardiovascular and related diseases) throughout the remainder of the paper.

Our primary objective was to present the level and variability in direct medical or programmatic costs for treating CVRD in LMICs. Our secondary objective was to identify gaps in knowledge and suggest future economic research needs to support policy development, resource allocation, and decision-making.

## Methods

### Search strategy and article retrieval

We conducted our review according to the PRISMA guidelines [14]. We searched the following databases: Medline, EMBASE, NHS-EED, HEED and EconLit. We adapted the search terms specifically to each database, using 1) terms related to cardiovascular diseases and risk conditions plus 2) terms for LMICs and regions plus 3) terms denoting economic evaluation. Additional file 1 details the full strategy. We limited our results to articles published on or after January 1^st^, 2000 until approximately July 1^st^, 2014. We screened eligible titles and abstracts to determine which articles would be included for final review. Two independent reviewers reviewed the full text articles. In addition, we examined the references of the included articles and prior reviews for potentially relevant studies. Broadly, the following criteria were used to determine inclusion:

- Included an economic evaluation
- Included evaluation of at least one low- or middle-income country (as defined by the World Bank)
- Referred to our previously standardized list of cardiovascular or related-related conditions
- Made available in English
- Published on or after January 2000.

We used both the Drummond Checklist and Mogyorosy and Smith’s 2012 literature review for guidance in creating the following guidelines regarding quality and inclusion [15, 16]:

- The study must have conducted at least one type of economic evaluation, including cost analysis, cost-effective analysis, or cost-utility analysis, with clearly presented unit cost data.
- We did not consider costs with health outcomes as a denominator, such as disability-adjusted life years (DALYs), quality adjusted life years (QALYs), or life-years saved (LYS).
- The study must have utilized either original unit cost data regarding a CVRD intervention delivered in a health systems setting, or unit costs from a credible and known source, such as WHO-CHOICE. We did not consider population-level interventions or policy costs.
- The study must have presented direct intervention costs (medical or programmatic) from the provider perspective, regardless of payer or indirect costs. Studies that considered multiple perspectives were only considered if they clearly delineated provider costs. Provider costs were defined as those delivering the services, such as those incurred by health systems, hospitals or clinics, governmental bodies, funders, or programs.
- The study had to meet certain quality standards, which includes having a description of the intervention and analysis, detailing the time and location of data collection, and clearly stating the year and currency of presented costs.

**Fig. 1:**
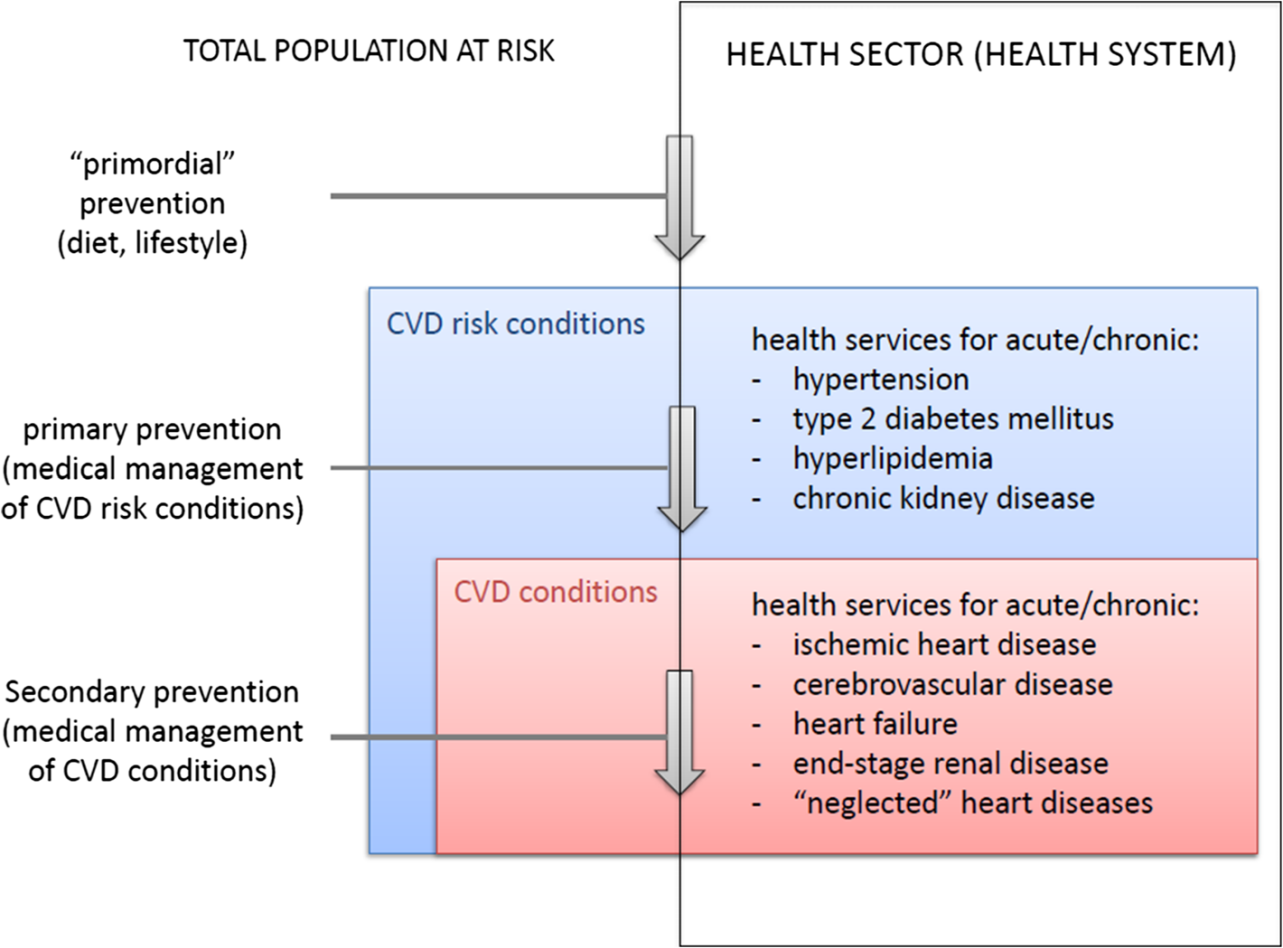
CVRD Conceptual Framework. This framework informed our inclusion criteria and subsequent data extraction and organization

To inform the organization and synthesis of the literature across conditions and interventions, we created a conceptual framework of the clinical and public health considerations in CVD treatment (Fig. 1). There are several points at which one can intervene on cardiovascular risk factors, risk conditions, and CVDs themselves, both within and outside of the health system. This review examines only the costs that exist within the health sector, and health facilities specifically, but across the spectrum of cardiovascular risk conditions and diseases themselves.

### Data extraction

For each eligible study we entered the following information into an electronic database: condition(s), country or region, target population, type of treatment or intervention, and level of care. We also noted study methods, such as sample size and strategy, exchange rate, discount rate, sensitivity analysis, and cost categories. The total treatment or intervention costs, defined as the total cost per patient, were extracted in their original currency and year. For acute events, we used the total cost per patient as presented. For recurring costs, such as hemodialysis treatments or ongoing hypertension management, we calculated the cost per treatment or per year. Differences in inpatient and outpatient costs were noted where reported and applicable. Of note, most of the included studies reported multiple costs, particularly if they discussed more than one CVRD intervention or more than one geographic setting.

We converted all costs to US Dollars (2012), the currency and year in which we present all costs in this paper. When possible, we extracted the data in local currency units (LCUs), inflated it to 2012 rates using the World Bank consumer price indices, and converted it to 2012 US Dollars via World Bank rates. When costs were presented for regions, we used the largest LMIC country in the region as a proxy. For data presented in international dollars, we converted it back into LCUs using World Bank Purchasing Power Parity, then we followed our standard processes. Once the data were in a common currency, we grouped similar interventions and qualitatively compared the magnitudes and variability of total and input costs. We combined data into the two overarching groups to reflect our aforementioned framework: management of CVD risk conditions and treatment of acute and chronic CVDs. The former group included hypertension, hyperlipidemia, CKD, T2DM, and generic CVD prevention. The latter group focused specifically on ischemic heart disease, non-ischemic heart diseases, stroke, heart failure, T2DM sequelae (specifically, microvascular complications such as foot ulcers and retinopathy), and end-stage renal disease (ESRD), which is due predominately to poorly controlled hypertension and T2DM.

## Results

**Fig. 2:**
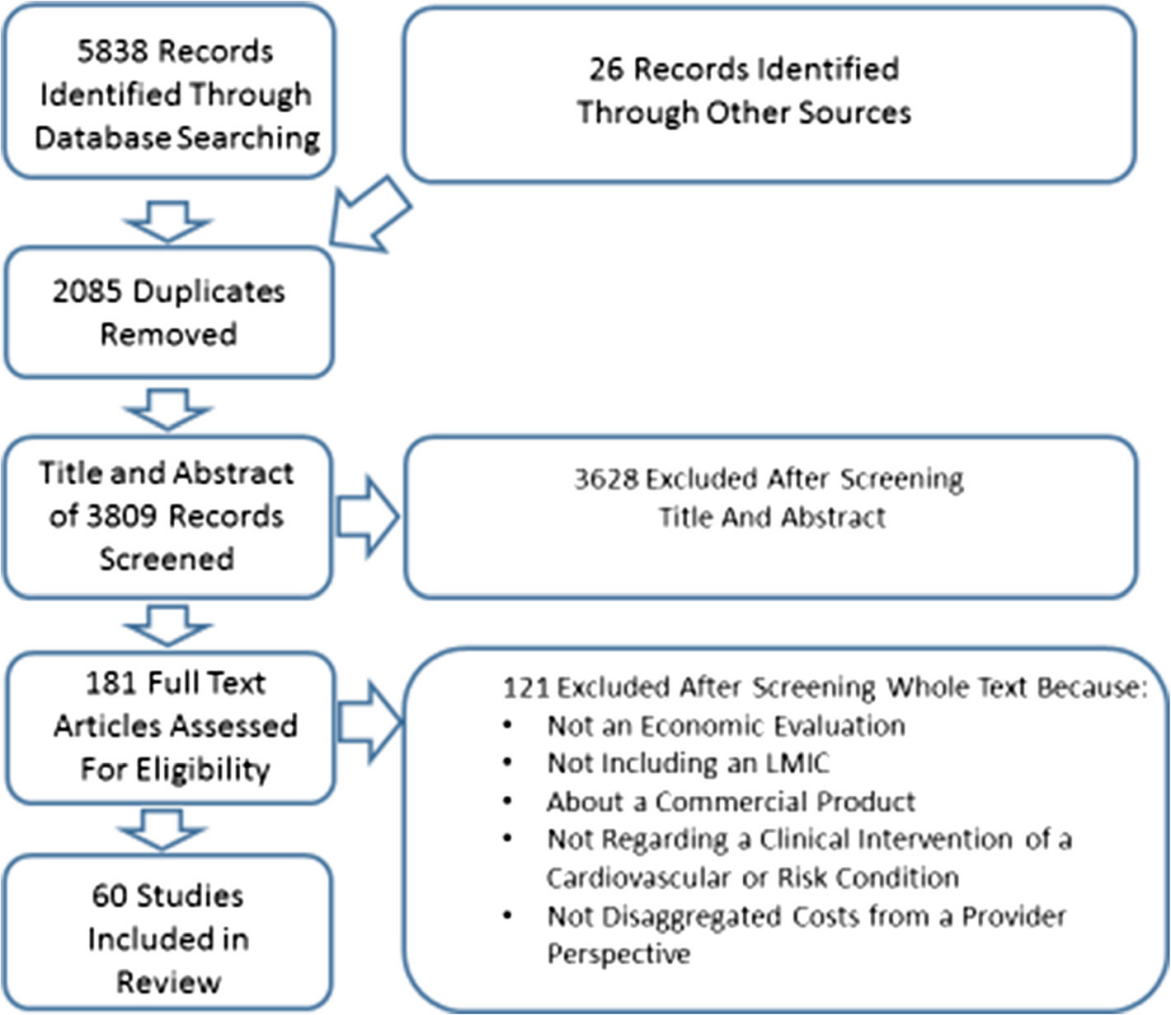
Article Selection Flow Chart (PRISMA STANDARDS). Flow diagram for the selection of published articles evaluating the costs of providing preventive care or treatment for cardiovascular disease, diabetes, and chronic kidney disease in low- and middle-income countries

The results of the systematic search process can be found in Fig. 2. 60 studies met our final inclusion criteria, presenting approximately 143 unit costs reported in Table 1. An additional 121 studies were reviewed at the full text stage but were excluded on the basis of our inclusion criteria or for methodological reasons, i.e., they did not meet the criteria in our quality checklist.

**Table 1:** Unit cost data for the treatment of cardiovascular risk conditions (USD 2012)

	Sub-Condition (If Applicable)	Intervention or Treatment	Country	Cost Presented in Paper	Unit	Currency (Year)	Cost in USD (2012)	Unit
Management of cardiovascular risk conditions								
Hypertension								
		Hypertension Drug Treatment [34]	Nigeria	300.00–2,100.00	Per month	Nigeria (2010)	$2.38–$16.66	Per patient per year
		Pharmacological high blood pressure and cholesterol treatment [35]	Tanzania	1.57–54.28	Per year	US (2005)	$2.21–$76.29	Per patient per year
		Pharmacological high blood pressure treatment [33]	Argentina	49.72	Per patient per year	US (2007)	$52.24	Per patient per year
		Community Hypertension Control Program: Home Health Education and General Practitioner [36]	Pakistan	3.99	Per patient per year	US (2007)	$4.96	Per patient per year
		Community Hypertension Control Program: Home Health Education [36]	Pakistan	3.34	Per patient per year	US (2007)	$4.15	Per patient per year
		Community Hypertension Control Program: General Practitioner [36]	Pakistan	0.65	Per patient per year	US (2007)	$0.81	Per patient per year
		Community Hypertension Reduction Program (1-year) [37]	China	7.17	Per patient per year	US (2009)	$8.67	Per patient per year
		Guideline-Oriented Training Program for Hypertension Control in Community Health Centers [38]	China	79.30	Per year	US (2002)	$138.96	Per year
		Hypertension Outpatient Treatment [32]	South Africa	86.00	Per patient per year	US (2001)	$169.28	Per patient per year
		Hypertension Outpatient Treatment [39]	China	487.30	Per patient per year	US (2010)	$565.54	Per patient per year
		Hypertension Outpatient Treatment at Urban Health Clinic [40]	Thailand	916.54	Per patient per year	Thailand (1999)	$41.42	Per patient per year
		Hypertension Outpatient Treatment [41]	China	96.90	Per outpatient visit	China (2003)	$20.28	Per outpatient visit
		Hypertension Outpatient Treatment and Medications [42]	Tanzania	38.00	Per patient per year	US (2012)	$38.00	Per patient per year
		Hypertension Inpatient Visit [41]	China	3,904.00	Per inpatient stay	China (2003)	$817.10	Per inpatient stay
		Hypertensive Emergency Treatment Cost (Inpatient and Outpatient) [43]	Congo, Rep.	159,600.00	Per patient	Congo, Rep. (2006)	$400.90	Per patient
Hyperlipidemia								
		Screening for dyslipidemia among healthy 35–39 year olds [44]	Thailand	127.22	Per patient screened	Thailand (2008)	$4.48	Per patient screened
		Screening for dyslipidemia among healthy 35–60 year olds [45]	Thailand	1,043.60	Per case detected	Thailand (2008)	$36.77	Per case detected
		Pharmacological high cholesterol treatment [33]	Argentina	118.79	Per patient per year	US (2007)	$124.81	Per patient per year
		Home visit by Health Care Professional [40]	Thailand	574.86	Per visit	Thailand (1999)	$25.98	Per visit
Chronic Kidney Disease								
		Amlodipine (Anti-hypertensive Drug Therapy for Patients with Diabetes, Hypertension, and Nephropathy) [29]	China	2,013.00	Per year	US (2004)	$3,356.78	Per patient per year
		Irbesartan (Anti-hypertensive Drug Therapy for Patients with Diabetes, Hypertension, and Nephropathy) [29]	China	1,660.00	Per year	US (2004)	$2,768.13	Per patient per year
		Amlodipine (Anti-hypertensive Drug Therapy for Patients with Diabetes, Hypertension, and Nephropathy) [29]	Malaysia	332.00	Per year	US (2004)	$503.07	Per patient per year
		Irbesartan (Anti-hypertensive Drug Therapy for Patients with Diabetes, Hypertension, and Nephropathy) [29]	Malaysia	258.00	Per year	US (2004)	$390.94	Per patient per year
		Amlodipine (Anti-hypertensive Drug Therapy for Patients with Diabetes, Hypertension, and Nephropathy) [29]	Thailand	779.00	Per year	US (2004)	$1,302.06	Per patient per year
		Irbesartan (Anti-hypertensive Drug Therapy for Patients with Diabetes, Hypertension, and Nephropathy) [29]	Thailand	1,340.00	Per year	US (2004)	$2,239.75	Per patient per year
Type 2 Diabetes Mellitus								
		Screening using fasting capillary blood glucose [46]	China	10.00	Per test	China (2009)	$1.77	Per patient
		Screening using Diabetes Risk Score [46]	China	5.00	Per test	China (2009)	$0.89	Per patient
		Screening for diabetes in a community health clinic [47]	China	3.00	Per patient	US (2007)	$4.25	Per patient
		Tertiary Hospital Diagnostic Test [46]	China	95.00	Per test	China (2009)	$16.82	Per patient
		Primary Care - Behavior management program to prevent diabetes [48]	India	54.67	Per patient per year	US (2006)	$78.93	Per patient per year
		Primary Care - Metformin regimen to prevent diabetes [48]	India	53.00	Per patient per year	US (2006)	$76.53	Per patient per year
		Primary Care - Behavior management program with Metformin regime to prevent diabetes [48]	India	69.67	Per patient per year	US (2006)	$100.59	Per patient per year
		Mean total annual direct medical cost (without complication) [49]	China	5,313.20	Per year	China (2007)	$989.16	Per patient per year
		Mean total annual direct medical cost (with complication) [49]	China	13,320.10	Per year	China (2007)	$2,479.82	Per patient per year
		Annual treatment costs (inpatient and outpatient costs) [50]	Thailand	6,331.00	Per patient per year	Thailand (2001)	$277.14	Per patient per year
		Annual treatment costs (inpatient and outpatient costs) [51]	Nigeria	47,924.36	Per patient per year	Nigeria (2010)	$380.14	Per patient per year
		Annual Diabetes Treatment in Community Clinic (Including Inpatient and Outpatient Visits) [52]	Brazil	1,319.15	Per year	US (2010)	$1,335.52	Per patient per year
		Annual treatment costs (inpatient and outpatient costs) [53]	India	3,006.00	Per patient per year	India (2012)	$56.25	Per patient per year
		Urban Health Center providing primary community care [40]	Thailand	1,408.59	Per patient per year	Thailand (1999)	$63.66	Per outpatient visit
		Inpatient stay w/o hypertension (Average 7 Days LOS) [54]	India	18,650.00	Per inpatient stay	India (2007)	$558.77	Per inpatient stay
		Inpatient Stay w/hypertension (Average 5 Days LOS) [54]	India	21,000.00	Per inpatient stay	India (2007)	$629.18	Per inpatient stay
		Inpatient stay w/o hypertension (Average 7 Days LOS) [54]	India	28,000.00	Per two years	India (2007)	$419.45	Per patient per year
		Inpatient Stay w/hypertension (Average 5 Days LOS) [54]	India	38,000.00	Per two years	India (2007)	$569.25	Per patient per year
		Inpatient Treatment: without complication (Average 9.8 Days LOS) [55]	China	6,903.93	Per patient	China (2006)	$1,346.37	Per inpatient stay
		Inpatient Treatment: with chronic complications (Average 9.8 Days LOS) [55]	China	7,193.15	Per patient	China (2006)	$1,402.77	Per inpatient stay
		Inpatient/Hospital Admission [56]	Thailand	95.99	Per day	US (2008)	$112.65	Per day
		Inpatient Treatment in Community Clinic (No LOS Stated) [52]	Brazil	26.32	Per year	US (2010)	$26.65	Per patient per year
		Outpatient Care [56]	Thailand	3.94	Per outpatient visit	US (2008)	$4.63	Per outpatient visit
		Outpatient Care in Community Clinic [52]	Brazil	1,216.33	Per year	US (2010)	$1,231.43	Per patient per year
		Outpatient treatment for patient without complications [57]	India	4,493.00	Per year	India (2009)	$112.05	Per inpatient stay
		Outpatient Treatment [58]	Nepal	16.95	Per outpatient visit	US (2010)	$17.45	Per outpatient visit
		Outpatient Treatment [58]	Nepal	130.52	Per year	US (2010)	$134.38	Per patient per year
		Outpatient Treatment [59]	Brazil	1,014.00	Per patient per year	US (2007)	$1,322.64	Per patient per year
		Outpatient Treatment [60]	Pakistan	1,468.90	Per outpatient visit	Pakistan (2006)	$32.34	Per outpatient visit
		Outpatient Treatment [41]	China	163.80	Per outpatient visit	China (2003)	$34.28	Per outpatient visit
Other clinical management of CVD risk conditions								
	Generic primary prevention	Modified poly-pill strategy [33]	Argentina	103.46	Per patient per year	US (2007)	$108.70	Per patient per year
		General Outpatient Visit [40]	Thailand	67.82	Per outpatient visit	Thailand (1999)	$3.07	Per outpatient visit
		Bupropion treatment HCW counseling for tobacco cessation [33]	Argentina	117.15	Per patient per year	US (2007)	$123.09	Per patient per year
	Rheumatic Fever	Prevention of Rheumatic Fever (throat culture)	India	1,088.56	Per patient	India (2007)	$32.61	Per patient
		Post rheumatic fever prophylaxis [61]	India	879.35	Per patient	India (2007)	$26.35	Per patient
		Inpatient Treatment for acute rheumatic fever [62]	South Africa	2,958.00	Per patient	US (2010)	$2,927.41	Per patient
		Echocardiographic screening for rheumatic heart disease in schoolchildren [63]	Fiji	2.07	Per patient screened	US (2008)	$2.27	Per patient screened

**Fig. 3:**
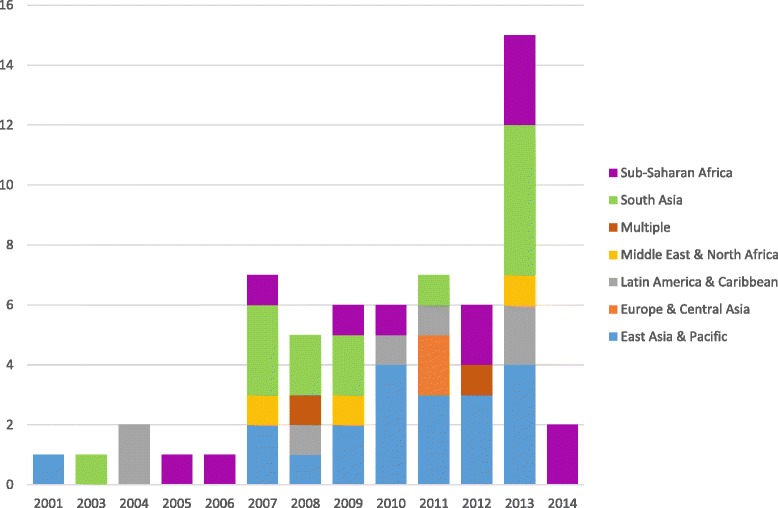
Articles Published by Year & Region. Figure shows the number of articles identified by our study disaggregated by the year in which they were published and the region (as defined by the World Bank) for which they provide data. Studies providing data for more than one region were categorized as “multiple”

The number of published articles for CVD costs in LMICs has increased dramatically since 2000, as seen in Fig. 3. In fact, we identified only five articles in the years 2000–2006, increasing to 16 articles published in 2013, the last complete year of our review. Most of the cost data occurred either in urban areas or at the national level of middle income countries, with only four studies considering low-income countries. The sample is mainly from Asian countries (34 articles), dominated by studies from India (8 articles) and China (12 articles). Almost a quarter of the cost data came from China; India contributed the next highest frequency of cost data (14 %). One-third of our cost data concerned diabetes, which was a considerably higher proportion than any other condition, and two-thirds of those diabetes costs were from Asian-based studies.

We obtained cost data from a variety of economic evaluations. Almost two-thirds of the articles (*n* = 37) were cost analyses, meaning they only collected and presented data on costs, not health outcomes. Thirty studies determined the total costs of interventions by reviewing hospital records while 29 studies used an ingredients approach, taking the sum cost of activities and resources used to find the total costs. Only one study used a questionnaire to collect data. Of the 60 studies, over half converted their costs in US Dollars or the hypothetical currency “International Dollars” for presentation (32 articles). Although it is difficult to measure the quality of a study objectively, economic evaluation guidelines recommend discounting future costs and conducting sensitivity analyses when assumptions are made. We noted that approximately one-third of the 60 studies conducted sensitivity analysis, and one-fourth used discounting in their methods; 11 articles used both.

### Management of cardiovascular risk conditions

Our review identified 60 cost estimates from 31 articles for clinical management of CVD risk conditions such as hypertension, hyperlipidemia, chronic kidney disease and T2DM). There were also brief mentions of pharmacological tobacco cessation measures, rheumatic fever and generic CVD prevention strategies such as polypill use. More than three-quarters of this data came from Asian countries, and more than half from China, India or Thailand.

We found 14 unit costs for hypertension management in LMICs, with drug therapy ranging from $2.21–$76.29. Using general practitioners in community programs cost only $0.81–$8.67 per patient per year, compared to annual outpatient costs to manage hypertension, which ranged from $38.00 to $565.54 per patient. Only three studies mentioned the costs of managing dyslipidemia, which were similar to managing hypertension. One study looked at the costs of managing chronic kidney disease across China, Malaysia and Thailand, with China being more expensive than Malaysia by a scale of six to eight. We identified 30 unit costs for managing diabetes. Two studies produced four costs on diagnosing diabetes, the most expensive of which were based in tertiary hospitals. Seven studies presented ten annual per patient costs of managing diabetes in a clinical setting, which ranged from $56.25 in India to $2,479.82 in China; the costs were higher in cases with complications and sequelae. Nine studies also presented 16 costs on inpatient and outpatient treatment for diabetes. Inpatient stays per bed-day ranged from $59.92–$143.14 with an average length of stay of around 7 days. An outpatient visit ranged from $4.63–$34.28, and we also found a ten-fold variation in yearly outpatient costs.

### Treatment and management of acute and chronic cardiovascular conditions

Our review also identified 83 cost estimates from 34 studies regarding the treatment of ischemic heart disease (IHD), stroke, heart failure, non-ischemic heart diseases, T2DM sequelae, and end-stage renal disease (ESRD).

Treatment of IHD typically consists of diagnostic procedures, surgical or other advanced medical procedures, and post-operative and long-term outpatient care. We found 8 articles analyzing IHD treatment with a total of 18 unit costs. An inpatient visit averaged $8,800, with a range from $455 to $22,500. The highest inpatient costs were associated with longer hospitalization periods, more severe conditions, and surgical interventions. The costs of surgical procedures, including catheter-based procedures (stenting and angioplasty) or coronary artery bypass surgery (CABG), ranged from $4,000–$22,000 per patient per procedure depending on the diagnostics, the complexity of the procedure, and whether medical therapy and recovery costs were included in the total cost estimates.

Our search returned 15 articles and 24 unit costs for both ischemic stroke (IS) and hemorrhagic stroke (HS), though many studies did not specify the type of stroke. In most settings, IS is the more common of the two, is generally easier to treat, and has a lower case-fatality rate. Four articles compared the costs HS and IS treatment [17–20]. Hemorrhagic strokes were generally more severe, required longer hospital stays, and involved surgical or intensive care unit costs; HS treatment costs were between 0 % and 50 % higher than IS treatment. The majority of articles provided the inpatient cost per stroke event, but did not distinguish the type of strokes. The average inpatient cost of a stroke was $3,240, but this varied widely by geographic setting, technical resources used, and average length of stay.

Five studies reported costs of T2DM sequelae, namely, diabetic foot ulcers and retinopathy. Severity of these impairments and costs saved by screening drove the total costs of both, though in opposite directions [21–25]. For instance, screening for retinopathy was generally below $30, while diabetic foot ulcers ranged from less than $1 to over $7,000 in the cases of infection and amputation.

Five studies reported costs of end-stage renal disease (ESRD), primarily long-term dialysis or renal transplantation. The cost of one session of hemodialysis for ESRD ranged $79 to $97 [26–28], while one study estimated average annual dialysis costs at $61,000 in Southeast Asia [29]. Kidney transplantation and post-operative care ran from $5,000 to $21,000 in the Sudan and Iran, respectively [28, 30].

Other types of CVD, such as heart failure, Chagas cardiomyopathy (CC), congenital heart disease, and rheumatic heart disease, are important contributors to the burden of NCDs in LMICs. However, we identified only a handful of studies on the cost of treating these conditions (Table 2).

**Table 2:** Unit cost data for the treatment of cardiovascular disease and related condition (USD 2012)

Treatment and Management of Acute/Chronic Cardiovascular and Related Conditions								
Ischemic Heart Disease								
		Outpatient Visit (All direct medical costs) [41]	China	245.40	Per outpatient visit	China (2003)	$51.36	Per outpatient visit
		CABG Procedure (Procedure and Medical Therapy) [32]	South Africa	11,431.00	Per patient	US (2001)	$22,500.46	Per patient
		CABG Procedure (Procedure only, no medical therapy) [64]	China	7,300.00	Per patient	US (2007)	$10,339.01	Per patient
		CABG Procedure (Procedure and Medical Therapy) [65]	Armenia	3,368.19	Per patient	US (2005)	$5,546.46	Per patient
		CABG Procedure (Procedure and Medical Therapy) [66]	India	8,055.00	Per patient	US (2006)	$11,630.62	Per patient
		Stenting Procedure (Procedure only, no medical therapy) [64]	China	10,000.00	Per patient	US (2007)	$14,163.03	Per patient
		Catheter-based revascularization Inpatient Treatment [32]	South Africa	4,737.00	Per patient	US (2001)	$9,324.18	Per patient
		Total operative procedures and examinations [67]	Brazil	444.29	Per patient	Brazil (2001)	$455.47	Per patient
		Coronary Angioplasty Diagnostic Strategy Including Therapy [64]	China	3,568.00	Per patient	US (2007)	$5,053.37	Per patient
		Computed Tomography Angiography and Coronary Angioplasty Diagnostic Strategy [64]	China	2,971.00	Per patient	US (2007)	$4,207.84	Per patient
		Inpatient Visit (All direct medical costs) [33]	Argentina	4,245.39	Per patient	US (2007)	$4,460.54	Per patient
		Inpatient Visit (All direct medical costs) [41]	China	11,008.20	Per inpatient stay	China (2003)	$2,304.00	Per inpatient stay
		Inpatient Visit (All direct medical costs) [68]	Vietnam	31,400,000.00	Per inpatient stay	Vietnam (2005)	$3,257.15	Per inpatient stay
		Average Inpatient Treatment (All direct medical costs) [32]	South Africa	5,636.00	Per patient	US (2001)	$11,093.74	Per patient
		Additional cost if a CABG or valve replacement surgery results in a hospital-acquired infection [66]	India	14,818.00	Per patient	US (2006)	$21,395.72	Per patient
		Post CHD with CABG (1st year) [32]	South Africa	1,300.00	Per year	US (2001)	$2,558.88	Per patient per year
		Post CHD with CABG (subsequent years) [32]	South Africa	600.00	Per year	US (2001)	$1,181.02	Per patient per year
		Post CHD without CABG (1st year) [32]	South Africa	1,500.00	Per year	US (2001)	$2,952.56	Per patient per year
		Post CHD without CABG (subsequent years) [32]	South Africa	840.00	Per year	US (2001)	$1,653.43	Per patient per year
Stroke								
	Hemorrhagic Stroke	Inpatient Visit for Stroke Care (Average 11.8 Days LOS) [17]	Turkey	1,348.00	Per patient	US (2007)	$1,444.46	Per patient
		Inpatient Visit for Stroke Care (Average 12 Days LOS) [18]	Brazil	1,831.00	Per patient	US (2007)	$2,388.33	Per patient
		Tertiary Treatment and Follow-Up - Coiling (6 months) [69]	Pakistan	304,800.00	Per patient	Pakistan (2007)	$6,236.79	Per patient
		Tertiary Treatment and Follow-Up - Endovascular clipping (6 months) [69]	Pakistan	187,620.00	Per patient	Pakistan (2007)	$3,839.06	Per patient
	Ischemic Stroke	Inpatient Visit for Stroke Care (Average 10.4 Days LOS) [17]	Turkey	956.00	Per patient	US (2007)	$1,024.41	Per patient
		Inpatient Visit for Stroke Care (Average 13.3 Days LOS) [18]	Brazil	1,645.00	Per patient	US (2007)	$2,145.71	Per patient
		Inpatient Visit for Stroke Care (Average 18.5 Days LOS) [70]	China	67.00	Per patient per day	US (2010)	$77.76	Per patient
		Inpatient Visit for Stroke Care (Average 18.5 Days LOS) [70]	China	983.00	Per patient	US (2010)	$1,140.84	Per patient
		Diagnostics and preventative care for high-risk patients at a tertiary hospital [71]	China	435.40	Per patient per year	US (2010)	$505.31	Per patient per year
	Stroke (Non-Specified)	Outpatient Visit [41]	China	264.80	Per outpatient visit	China (2003)	$55.42	Per outpatient visit
		Direct Medical Costs for Managing a Stroke Patient in Year Following Stroke [72]	Nigeria	62,217.00	Per patient	US (2012)	$62,217.00	Per patient
		Direct Medical and follow up (6-months) [73]	India	57,381.00	Per patient	India (2011)	$1,173.80	Per patient
		Inpatient Visit for Stroke Care: Average across all wards (3–5 Days Ave. LOS) [31]	Pakistan	1,179.00	Per patient	US (2001)	$2,160.99	Per patient
		Inpatient Visit for Stroke Care: ICU (3–5 Days LOS) [31]	Pakistan	3,583.50	Per patient	US (2001)	$6,568.20	Per patient
		Inpatient Visit for Stroke Care: Private Ward (3–5 Days LOS) [31]	Pakistan	1,248.00	Per patient	US (2001)	$2,287.46	Per patient
		Inpatient Visit for Stroke Care: General Ward (3–5 Days LOS) [31]	Pakistan	1,010.00	Per patient	US (2001)	$1,851.23	Per patient
		Inpatient Visit for Stroke Care (LOS not stated) [32]	South Africa	8,633.00	Per patient	US (2001)	$16,992.95	Per patient
		Inpatient Visit for Stroke Care (LOS not stated) [74]	Tanzania	138,000.00	Per patient	Tanzania (2006)	$160.18	Per patient
		Inpatient Visit for Stroke Care (LOS not stated) [33]	Argentina	3,455.48	Per patient	US (2007)	$3,630.60	Per patient
		Inpatient Visit for Stroke Care (Up to 3 Days LOS) [43]	Congo, Rep.	158,120.00	Per patient	Congo, Rep. (2006)	$397.19	Per patient
		Inpatient Visit for Stroke Care (LOS not stated) [41]	China	7,953.10	Per inpatient stay	China (2003)	$1,664.57	Per inpatient stay
		Inpatient Visit for Stroke Care: small hospital (Average 20 Days LOS) [20]	China	7,119.00	Per inpatient stay	China (2006)	$1,388.31	Per inpatient stay
		Inpatient Visit for Stroke Care: tertiary hospital (Average 20 Days LOS) [20]	China	12,344.00	Per inpatient stay	China (2006)	$2,407.26	Per inpatient stay
		Inpatient Visit for Stroke Care (Average 6.4 Days LOS) [19]	Malaysia	3,696.40	Per inpatient stay	Malaysia (2005)	$1,431.57	Per patient per admission
Heart Failure								
		Inpatient treatment for heart failure due to systolic or diastolic dysfunction with a Chagas’ cardiomyopathy diagnosis [75]	Brazil	467.00	Per day	Brazil (2006)	$324.23	Per day
		Outpatient Treatment [76]	Brazil	14.40	Per outpatient visit	Brazil (2002)	$13.61	Per outpatient visit
		Outpatient Treatment plus medications [76]	Brazil	557.28	Per year	Brazil (2002)	$526.79	Per patient per year
		Inpatient treatment for heart failure due to systolic or diastolic dysfunction with Non-Chagas’ cardiomyopathy (other etiologies) [75]	Brazil	308.00	Per day	Brazil (2006)	$213.84	Per day
		Inpatient Care (Up to 3 Days LOS) [43]	Congo, Rep.	81,900.00	Per patient	Congo, Rep. (2006)	$205.73	Per patient
		Inpatient Care (Average 6.5 Days LOS) [76]	Brazil	4,033.62	Per inpatient stay	Brazil (2002)	$3,812.90	Per inpatient stay
Neglected Heart Diseases								
	Congenital heart disease	Operations and treatments, including pre and post-operative care [67]	Brazil	1,428.05	Per patient	Brazil (2001)	$1,463.95	Per patient
	Rheumatic Heart Disease	Inpatient Treatment (Average 7 Days LOS) [62]	South Africa	1,597.00	Per patient	US (2010)	$1,580.49	Per patient
		Tertiary care (including inpatient care and surgery) [61]	India	1,547.17	Per patient	India (2007)	$46.35	Per patient
Type Two Diabetes Mellitus								
	Diabetic Foot Ulcers	Total treatment [21]	Pakistan	2,700.00	Per patient	Pakistan (2005)	64.15–$1165.65	Per patient
		Total Treatment (healed) [22]	China	1,673.00	Per patient	$Int (2010)	$585.60	Per patient
		Total Treatment (trans-tibial amputation) [22]	China	21,372.00	Per patient	$Int (2010)	$7,480.87	Per patient
		Total Treatment (healed) [22]	India	1,192.00	Per patient	$Int (2010)	$85.51	Per patient
		Total Treatment (trans-tibial amputation) [22]	India	19,599.00	Per patient	$Int (2010)	$1,405.97	Per patient
		Total Treatment (healed) [22]	Tanzania	102.00	Per patient	$Int (2010)	$0.24	Per patient
		Total Treatment (trans-tibial amputation) [22]	Tanzania	3,060.00	Per patient	$Int (2010)	$7.30	Per patient
		Total Treatment [23]	Nigeria	93,256.70	Per patient	Nigeria (2003)	$1,618.55	Per patient
	Retinopathy	Retinopathy screening in a primary care setting [24]	South Africa	22.00	Per patient	US (2007)	$26.10	Per patient
		Laser treatment in retinopathy confirmed cases [24]	South Africa	144.00	Per patient	US (2007)	$170.85	Per patient
		Screening Retinal examination at a hospital [25]	India	5.84	Per patient	US (2009)	$7.05	Per patient
		Screening single laser photocoagulation treatment at a hospital [25]	India	7.51	Per patient	US (2009)	$9.07	Per patient
		Screening Using Telescreening in rural areas [25]	India	7.36	Per patient	US (2009)	$8.88	Per patient
End-Stage Renal Disease								
		Dialysis [29]	China	56,584.00	Per year	US (2004)	$94,356.62	Per patient per year
		Dialysis [29]	Malaysia	19,054.00	Per year	US (2004)	$28,871.91	Per patient per year
		Dialysis [29]	Thailand	31,651.00	Per year	US (2004)	$52,903.20	Per patient per year
		1 session of hemodialysis in a hospital [27]	Jordan	72.00	Per session	US (2010)	$78.76	Per session
		Hemodialysis maintenance session in a hospital [26]	Iran, Islamic Rep.	52.60	Per session	US (2007)	$96.66	Per session
		Hemodialysis in a hospital [28]	Sudan	15,747.68	Per patient per year	Sudan (2009)	$8,374.01	Per patient per year
		Hemodialysis in a hospital [28]	Sudan	146.58	Per patient per session	Sudan (2009)	$77.95	Per patient per session
		Renal Transplant (first year expenses) [29]	China	54,886.00	Per year	US (2004)	$91,525.12	Per patient per year
		Maintenance post index year of transplant patient [29]	China	27,259.00	Per year	US (2004)	$45,455.73	Per patient per year
		Renal Transplant (first year expenses) [29]	Malaysia	70,022.00	Per year	US (2004)	$106,102.06	Per patient per year
		Maintenance post index year of transplant patient [29]	Malaysia	14,111.00	Per year	US (2004)	$21,381.94	Per patient per year
		Renal Transplant (first year expenses) [29]	Thailand	45,953.00	Per year	US (2004)	$76,808.34	Per patient per year
		Maintenance post index year of transplant patient [29]	Thailand	19,349.00	Per year	US (2004)	$32,340.97	Per patient per year
		Kidney transplantation, including operation and following year [28]	Sudan	34,097.85	Per patient	Sudan (2009)	$18,131.93	Per patient
		Kidney transplantation, after the first year [28]	Sudan	24,499.00	Per patient	Sudan (2009)	$13,027.63	Per patient
		Transplantation Procedure [30]	Iran, Islamic Rep.	2,048.00	Per patient	US (2005)	$4,769.20	Per patient
		Total cost (Transplant procedure, 1 year immunosuppression, donor costs) [30]	Iran, Islamic Rep.	9,224.00	Per patient	US (2005)	$21,480.05	Per patient

Definitions: Per capita costs were divided by the population of the study country in 2012 as defined by the World Bank. Primary prevention defined as actions to reduce the probability of initial occurrence of disease. Secondary prevention defined as actions following the occurrence of disease to prevent either the recurrence of the same event or to reduce the risk of a different but related event

*Abbreviations*: *CABG* Coronary Artery Bypass Grafting, *CHD* Coronary Heart Disease, *LOS* Length of Stay, *ICU* Intensive Care Unit

## Discussion

Treating CVD and its risk conditions is complex, due in part to the interrelationship between hypertension, diabetes, and ischemic heart disease, and the fact that multiple shared risk factors affect CVD health outcomes. The clinical heterogeneity of CVD can make treatment costs for a single condition much more variable than in the case of infectious diseases or some other chronic diseases; these factors also affect the mean and distribution of costs within and across similar conditions. For instance, CVD encompasses different types of heart and related diseases, such as hypertension, stroke, and heart failure, with different levels of severity, and associated care and management. There are numerous clinical protocols for treating complicated conditions, and treatment includes different combinations of drugs, diagnostics and imaging technologies, surgery and different requirements for inpatient care and follow-up visits, making comparisons between studies all but impossible. Additionally, there are large variations in clinical characteristics, capabilities, and practices among and within countries; it is possible to have a wide distribution of costs even within a hospital if they offer various levels of care, such as general, specialty, and intensive care wards [31].

Another factor affecting the variation in costs was differences in methodology used across studies. There was a lack of standard reporting across the studies, most notably failure to describe cost ingredients. When studies disaggregated costs, there were not clear and common categories for input or activity cost categories. In addition, for disaggregated input costs, it was unclear when activities included personnel in the costs. Many studies provided costs for imaging, diagnostic or therapeutic services, without indicating whether personnel costs were included. Surgery was a common category, but descriptions of what resources comprised surgery or surgical procedures were absent from the majority of the studies. Cost data was most informative when disaggregated into categories and inputs, allowing the reader to better understand heterogeneity and make more useful comparisons. We also noticed a lack of clinical protocol reporting in publications. Reporting on clinical protocols underlying the CVD interventions may help readers evaluate the comparability and transferability of costs estimates across studies, depending on the study objective.

**Fig. 4:**
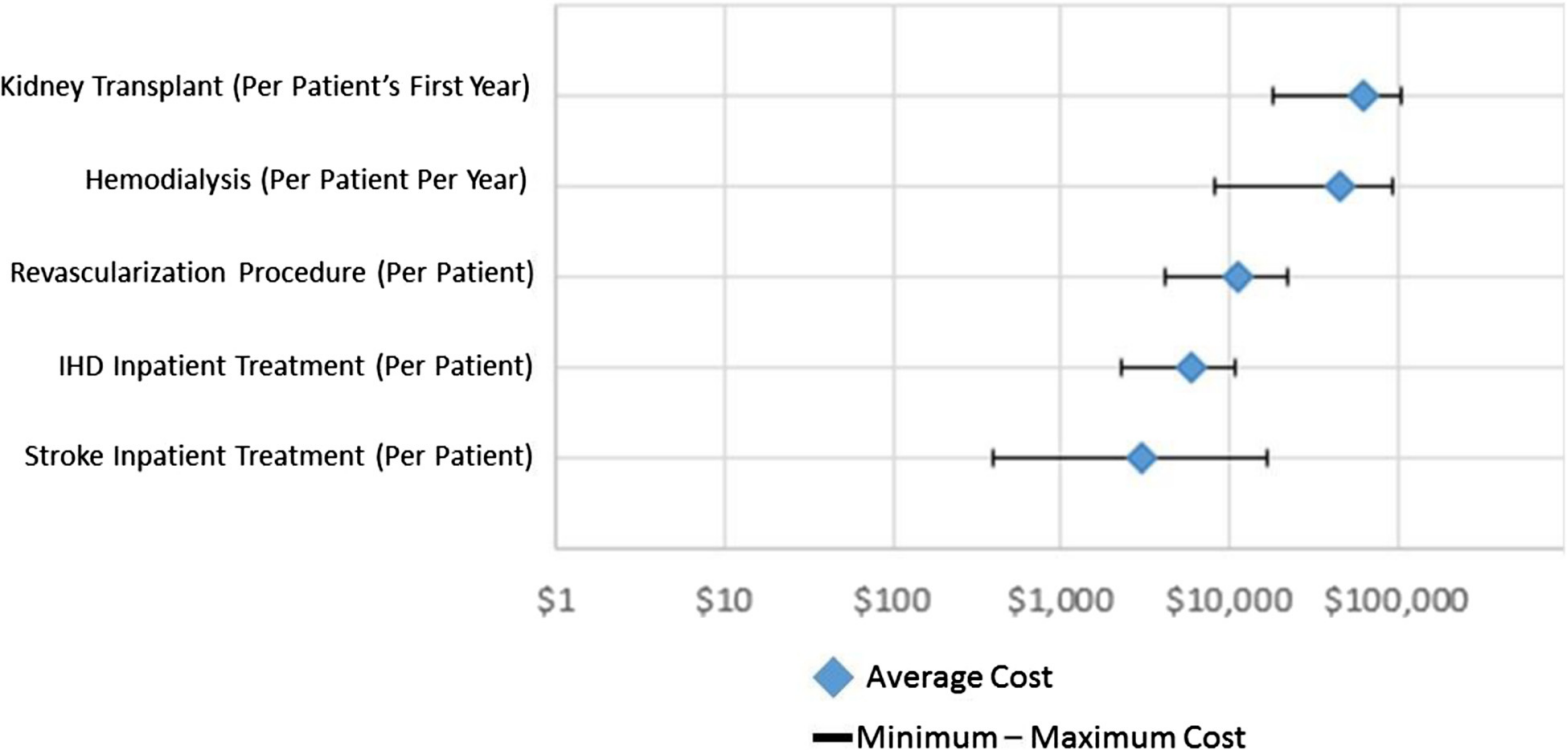
Hospital Costs by Condition (USD 2012). Showing the average, minimum, and maximum costs of inpatient treatment for various cardiovascular and chronic kidney disease conditions. Source: extracted cost data for this review

**Fig. 5:**
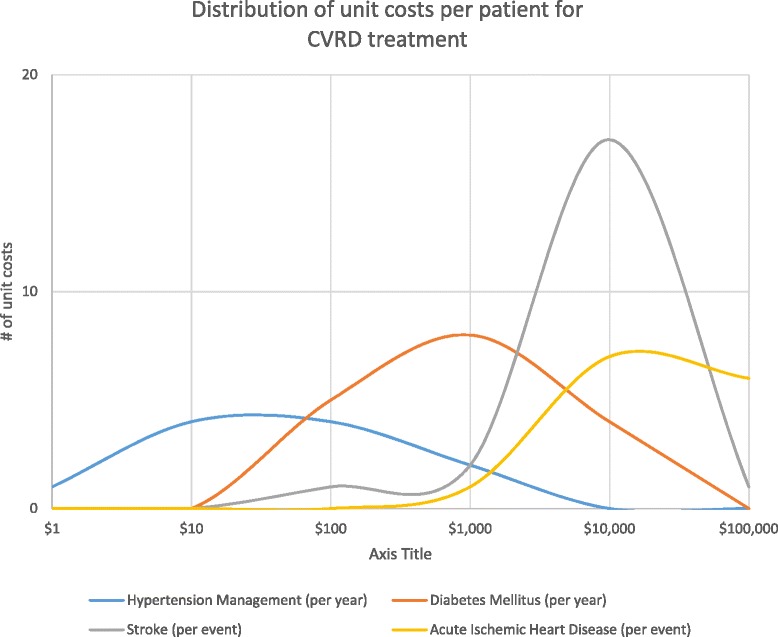
Unit Cost Distributions. Figure of four overlapping histograms showing the distribution of identified costs per patient for four conditions: hypertension management, diabetes mellitus, stroke, and acute ischemic heart disease. These four conditions had the most robust data available in the review

Trends in the data emerged despite these limitations. It is clear that there is growing interest and concern about the prevalence and cost of treating cardiovascular and risk conditions at every national income level. BRICS countries and a few other populous Asian countries produced about three-quarters of the literature we identified, laying a foundation of evidence for the costs of treating CVRDs in LMICs. The current evidence confirms that is expensive to provide treatment for complicated or advanced cardiovascular conditions, with inpatient treatment for CVD easily costing upwards of $10,000 per patient (Fig. 4). Looking at the distribution of costs by condition, managing risk conditions is generally much less expensive than treating acute CVD conditions (Fig. 5).

Our review identified critical gaps in information and demonstrated the substantial heterogeneity in treatment costs for CVRD across the available data for LMIC countries. The most obvious gap was the need for more consistent methodology, with clear presentation of data on cost ingredients and drivers. Cost data are used to inform resource allocation and to improve efficiency; health policy makers thus need to understand the underlying causes of health expenditures in order to make informed decisions. We identified no longitudinal studies in LMICs, however several modeled studies looked at health costs over a longer period of time. We anticipate that longitudinal studies would be particularly revealing regarding costs of chronic conditions, such as hypertension and diabetes, which require lifelong management. In the absence of longitudinal studies, health decision-makers are currently allocating CVD resources with an incomplete picture of future costs.

We found very few cost analyses of CVD conditions conducted in low-income countries. While these countries are likely the most heavily resource-constrained, they are also likely the least prepared to prevent and treat CVD conditions. We found no data on the cost of diabetes care in low-income countries. Middle-income countries, particularly upper-middle income countries such as Brazil, China, and South Africa, have produced research or publications regarding technologically complex interventions, revealing relatively higher health system capacity. As the double-burden of infectious and chronic conditions increases, and policy makers in low-income countries expand health resources for chronic conditions, published studies from other LMICs can provide economic evidence on CVD interventions.

Because our analysis focused on the unit costs of clinical interventions, we did not include many of the WHO Best Buys for NCDs such as tobacco taxation and regulation, population salt regulation, or health and physical activity promotion. Population based prevention measures typically cost pennies per person, but the total cost to the government will depend on the population size. It was beyond the scope of this paper to compare the total costs of population based interventions with clinical prevention and treatment costs. The evidence in this review strongly suggests that costs quickly escalate with advanced CVRD treatment; future investment in population prevention of CVRDs could reduce the need for these expensive clinical interventions.

We also excluded studies presenting the cost of implementing guidelines for initiating prevention strategies of CVRDs based on absolute risk [32, 33]. While prevention is extremely important for averting premature death and the high costs of surgery and post-operative care, setting risk guideline thresholds is less associated with changes in unit costs than with changes in long-term cost-effectiveness and total budget impact. The economic evaluations we identified regarding risk guidelines ultimately fell outside of our purview due to their health outcome denominators.

## Conclusion

The evidence for CVRD treatment and prevention costs in LMICs is limited, particularly in low-income countries. We recommend conducting economic studies alongside efforts to scale up treatment and preventions on CVRD in these settings. Initially standard methods of care or clinical protocol could be used to inform costs of CRVD treatment and prevention in order to estimate costs and their key drivers are across settings. There is also a need to estimate the actual resource use and costs in the provision of care, as such information can be used to improve efficiency and budgeting over time. In addition to following recommended costing analysis protocols [15], future research on the costs of CVD prevention and care should disaggregate the cost inputs, especially long term costs, since most individuals with CVRD experience worsening disease severity and more sequelae over time.

The burden of CVRD in LMIC is already substantial and will continue to increase in the absence of concerted prevention efforts, which could contain costs by reducing future spending on CVD. At the same time, the prevalence of CVD is already very high in regions such as Latin America and South and East Asia. Hence, efforts to allocate NCD resources equitably will inevitably include a balance of both prevention and advanced treatments for existing cases of CVD. Ongoing efforts to understand the cost of delivering CVRD care in these regions will be critical to achieving universal health coverage and improving overall population health.

## Additional files

Additional file 1: Full search report. (DOCX 21 kb)

## Abbreviations

CKD: Chronic Kidney Disease
CC: chagas cardiomyopathy
CVD: Cardiovascular disease
CVRD: Cardiovascular Disease and Risk Conditions
DALY: Disability-adjusted life-year
ESRD: End-stage renal disease
LYS: Life years saved
LCU: Local currency unit
LMIC: Low- and middle-income countries
NCD: Non-communicable disease
QALY: Quality-adjusted life-year
T2DM: Type two diabetes mellitus
WHO: World Health Organization
