# Supplementary material for: Provider costs for prevention and treatment of cardiovascular and related conditions in low- and middle-income countries: a systematic review
Source: BMC Public Health. 2015 Nov 26;15:1183. doi: 10.1186/s12889-015-2538-z (PMC4660724; doi:10.1186/s12889-015-2538-z)
Supplement: Additional file 1: — Full search report. (DOCX 21 kb) [file 12889_2015_2538_MOESM1_ESM.docx]

**Search report: cost-effectiveness CVD LMIC searches July 2014**

| **Search No.** | **Date** | **Database searched** | **Hits (before duplicate removal)** |
| --- | --- | --- | --- |
| 1 | 18/07/2014 | Medline (OVID) | 1982 |
| 2 | 26/06/2014 | EMBASE (OVID) | 3358 |
| 3 | 27/06/2014 | NHS-EED (Cochrane) | 156 |
| 4 | 27/06/2014 | HEED (Cochrane) | 19 |
| 5 | 27/06/2014 | Econlit (EBscoHost) | 353 |
| **FINAL NUMBER OF REFERENCES IN ENDNOTE AFTER DELETING DUPLICATES = 3809** | | | |

**Number of references in Endnote subgroups:**

- Econlit searches = 337
- Embase results = 1628
- Medline results = 1758
- NHS-EED = 86

**Search strategies:**

**MEDLINE and EMBASE (OVID)**

1 (CVD or secondary prevention or heart disease or cardiovascular disease or cardiometabolic disease or cardiometabolic disorder or cardio-metabolic disease or cardio-metabolic disorder or CABG).ab. or (CVD or secondary prevention or heart disease or cardiovascular disease or cardiometabolic disease or cardiometabolic disorder or cardio-metabolic disease or cardio-metabolic disorder or CABG).ti.

2 limit 1 to yr="2000 -Current"

3 (stent or coronary heart disease or coronary syndrome or heart failure or hypertensive or anti-hypertension or cholesterol or ischemia or atherosclerosis or hypertension or high blood pressure or blood pressure control or angiography or ischemic heart disease or acute myocardial infarction or coronary bypass surgery or cardiomyopathies or non-communicable disease or noncommunicable disease or NCDs or ventricular dysfunction or Angina or Acute coronary syndrome or cardiac output).ab. or (stent or coronary heart disease or coronary syndrome or heart failure or hypertensive or anti-hypertension or cholesterol or ischemia or atherosclerosis or hypertension or high blood pressure or blood pressure control or angiography or ischemic heart disease or acute myocardial infarction or coronary bypass surgery or cardiomyopathies or non-communicable disease or noncommunicable disease or NCDs or ventricular dysfunction or Angina or Acute coronary syndrome or cardiac output).ti.

4 limit 3 to yr="2000 -Current"

5 (ejection fraction or systolic failure or stroke or aneurysm or atherosclerosis or cerebrovascular disease or peripheral artery disease or peripheral vascular disease or diabetes or diabetic foot or blood sugar or Type 2 DM or T2DM or insulin resistance or impaired glucose tolerance or impaired fasting glycaemia or hyperlipidaemia or hyperlipidemia or dyslipidemia or chronic respiratory disease or COPD or Chronic obstructive pulmonary disease or chronic airway obstruction or asthma or restrictive lung disease or lung fibrosis or lung scarring or chronic bronchitis or bronchitis or kidney disease or hemodialysis or end stage renal disease or ESRD or renal transplant or kidney transplant).ab. or (ejection fraction or systolic failure or stroke or aneurysm or atherosclerosis or cerebrovascular disease or peripheral artery disease or peripheral vascular disease or diabetes or diabetic foot or blood sugar or Type 2 DM or T2DM or insulin resistance or impaired glucose tolerance or impaired fasting glycaemia or hyperlipidaemia or hyperlipidemia or dyslipidemia or chronic respiratory disease or COPD or Chronic obstructive pulmonary disease or chronic airway obstruction or asthma or restrictive lung disease or lung fibrosis or lung scarring or chronic bronchitis or bronchitis or kidney disease or hemodialysis or end stage renal disease or ESRD or renal transplant or kidney transplant).ti.

6 limit 5 to yr="2000 -Current"

7 (haemodyalisis or Retinopathy or Acute kidney injury or Renal disease or Dialysis or rheumatic fever or rheumatic heart disease or congenital heart disease or myocarditis or valvular heart disease or infective endocarditis or low salt or salt free or low sodium or sodium restricted or salt restricted or sodium free or salt reduction or smoking cessation or tobacco cessation or smoking ban or tobacco control or physical activity or exercise promotion or health promotion or dietary improvement or vegetable consumption or fruit consumption or obesity reduction or unhealthy diet or healthy diet or impaired glucose tolerance).ab. or (haemodyalisis or Retinopathy or Acute kidney injury or Renal disease or Dialysis or rheumatic fever or rheumatic heart disease or congenital heart disease or myocarditis or valvular heart disease or infective endocarditis or low salt or salt free or low sodium or sodium restricted or salt restricted or sodium free or salt reduction or smoking cessation or tobacco cessation or smoking ban or tobacco control or physical activity or exercise promotion or health promotion or dietary improvement or vegetable consumption or fruit consumption or obesity reduction or unhealthy diet or healthy diet or impaired glucose tolerance).ti.

8 limit 7 to yr="2000 -Current"

9 (physical education or (fruits and vegetables) or sugary beverage or body weight or body mass index or waist circumference or body fat or fat mass or abdominal fat or intra-abdominal fat or ectopic fat or subcutaneous fat or overweight or obesity or adiposity or adipose or BMI or waist-to hip or waist-hip ratio or alcohol reduction or alcohol regulation or alcohol restriction).ab. or (physical education or (fruits and vegetables) or sugary beverage or body weight or body mass index or waist circumference or body fat or fat mass or abdominal fat or intra-abdominal fat or ectopic fat or subcutaneous fat or overweight or obesity or adiposity or adipose or BMI or waist-to hip or waist-hip ratio or alcohol reduction or alcohol regulation or alcohol restriction).ti.

10 limit 9 to yr="2000 -Current"

11 ((early origin or maternal or gestational diabetes or birth weight or intra-uterine or infant feeding or breast feeding or child growth) and (Cardiometabolic or cardiovascular)).ab.

12 limit 11 to yr="2000 -Current"

13 ((Cardiometabolic or cardiovascular) and (early origin or maternal or gestational diabetes or birth weight or intra-uterine or infant feeding or breast feeding or child growth)).ti.

14 limit 13 to yr="2000 -Current"

15 ((task shifting or task-shifting or self management or self-management) and (cardiometabolic or cardiovascular)).ti.

16 limit 15 to yr="2000 -Current"

17 ((task shifting or task-shifting or self management or self-management) and (cardiometabolic or cardiovascular)).ab.

18 limit 17 to yr="2000 -Current"

19 ((change* or gain or loss or management or status or control) and weight).ab.

20 limit 19 to yr="2000 -Current"

21 2 or 4 or 6 or 8 or 10 or 12 or 14 or 16 or 18 or 20

22 (weight and (change* or gain or loss or management or status or control)).ti.

23 limit 22 to yr="2000 -Current"

24 21 or 23

25 (developing countries or developing country or (low and middle income countries) or LMIC or south America or latin America or Afghanistan or Albania or Algeria or Angola or Argentina or Armenia or Armenian or Azerbaijan or Bangladesh or Benin or Belize or Bhutan or Bolivia or Botswana or Brazil or Burkina Faso or Burundi or Cambodia or Khmer Republic or Kampuchea or Cameroon or Cameroons or Cameron or Camerons or Cape Verde or Central African Republic or Chad or China or Colombia or Comoros or Comoro Islands or Comores or Mayotte or Congo or Zaire or Costa Rica or Cote d'Ivoire or Ivory Coast or Djibouti or French Somaliland or Dominica or Dominican Republic or East Timor or East Timur or Timor Leste or Ecuador or Egypt or United Arab Republic or El Salvador or Eritrea or Ethiopia or Fiji or Gabon or Gabonese Republic or Gambia or Gaza or Georgia or Ghana or Grenada or Guatemala or Guinea or Guiana or Guyana or Haiti or Honduras or India or Maldives or Indonesia or Kenya or Kiribati or Lao PDR or Laos or Lesotho or Basutoland or Liberia or Libya or Madagascar or Malagasy Republic or Sabah or Sarawak or Malawi or Nyasaland or Mali or Marshall Islands or Mauritania or Mauritius or Agalega Islands or Mexico or Micronesia or Moldova or Moldovia or Moldovian or Mongolia or Montenegro or Morocco or Ifni or Mozambique or Myanmar or Myanma or Burma or Namibia or Nepal or Netherlands Antilles or Nicaragua or Niger or Nigeria or Muscat or Pakistan or Palau or Palestine or Panama or Paraguay or Peru or Philippines or Philipines or Phillipines or Phillippines or Rwanda or Ruanda or Nevis or Saint Lucia or St Lucia or Saint Vincent or St Vincent or Grenadines or Samoa or Samoan Islands or Navigator Island or Navigator Islands or Sao Tome or Senegal or Serbia or Montenegro or Seychelles or Sierra Leone or Sri Lanka or Ceylon or Solomon Islands or Somalia or Sudan or Suriname or Surinam or Swaziland or Tajikistan or Tadzhikistan or Tadjikistan or Tadzhik or Tanzania or Thailand or Togo or Togolese Republic or Tonga or Tunisia or Turkey or Turkmenistan or Turkmen or Uganda or Ukraine or Vanuatu or New Hebrides or Venezuela or Vietnam or Viet Nam or Zambia or Zimbabwe or Africa, Northern or Northern Africa or North Africa or Africa South of the Sahara or sub-Saharan Africa or subsaharan Africa or Africa, Central or central Africa or Africa, Eastern or Eastern Africa or east Africa or Africa, Southern or southern Africa or Africa, Western or western Africa or west Africa or Caribbean Region or Caribbean or Central America or Panama Canal Zone or French Guiana or Borneo or Mekong Valley or mekong delta or Republic of Congo or Congo-Brazzaville or Democratic Republic of the Congo or DRC or Congo-Kishasha or South Sudan or South Africa or Guinea-Bissau).ti.

26 limit 25 to yr="2000 -Current"

27 24 and 26

28 (cost or cost analysis or economics or cost savings or cost of illness or health care costs or direct service costs or drug costs or hospital costs or health expenditures or cost effectiveness or cost-effectiveness or cost of treatment or cost of disease or cost of care or health care cost or economic evaluation or cost analysis or economic analysis or cost benefit analysis or cost allocation or cost of services or medicine cost or hospital cost or out-of-pocket or expenses or expenditure or QALY or DALY).ti. or (cost or cost analysis or economics or cost savings or cost of illness or health care costs or direct service costs or drug costs or hospital costs or health expenditures or cost effectiveness or cost-effectiveness or cost of treatment or cost of disease or cost of care or health care cost or economic evaluation or cost analysis or economic analysis or cost benefit analysis or cost allocation or cost of services or medicine cost or hospital cost or out-of-pocket or expenses or expenditure or QALY or DALY).ab.

29 limit 28 to yr="2000 -Current"

30 27 and 29

**Cochrane Library**

#1 stent or coronary heart disease or coronary syndrome or heart failure or hypertensive or anti-hypertension or cholesterol or ischemia or atherosclerosis or hypertension or high blood pressure or blood pressure control or angiography or ischemic heart disease or acute myocardial infarction or coronary bypass surgery or cardiomyopathies or non-communicable disease or noncommunicable disease or NCDs or ventricular dysfunction or Angina or Acute coronary syndrome or cardiac output:ti,ab,kw Publication Year from 2000 to 2014 (Word variations have been searched)

#2 CVD or secondary prevention or heart disease or cardiovascular disease or cardiometabolic disease or cardiometabolic disorder or cardio-metabolic disease or cardio-metabolic disorder or CABG:ti,ab,kw Publication Year from 2000 to 2014 (Word variations have been searched)

#3 ejection fraction or systolic failure or stroke or aneurysm or atherosclerosis or cerebrovascular disease or peripheral artery disease or peripheral vascular disease or diabetes or diabetic foot or blood sugar or Type 2 DM or T2DM or insulin resistance or impaired glucose tolerance or impaired fasting glycaemia or hyperlipidaemia or hyperlipidemia or dyslipidemia or chronic respiratory disease or COPD or Chronic obstructive pulmonary disease or chronic airway obstruction or asthma or restrictive lung disease or lung fibrosis or lung scarring or chronic bronchitis or bronchitis or kidney disease or hemodialysis or end stage renal disease or ESRD or renal transplant or kidney transplant:ti,ab,kw Publication Year from 2000 to 2014 (Word variations have been searched)

#4 haemodyalisis or Retinopathy or Acute kidney injury or Renal disease or Dialysis or rheumatic fever or rheumatic heart disease or congenital heart disease or myocarditis or valvular heart disease or infective endocarditis or low salt or salt free or low sodium or sodium restricted or salt restricted or sodium free or salt reduction or smoking cessation or tobacco cessation or smoking ban or tobacco control or physical activity or exercise promotion or health promotion or dietary improvement or vegetable consumption or fruit consumption or obesity reduction or unhealthy diet or healthy diet or impaired glucose tolerance:ti,ab,kw Publication Year from 2000 to 2014 (Word variations have been searched)

#5 physical education or (fruits and vegetables) or sugary beverage or body weight or body mass index or waist circumference or body fat or fat mass or abdominal fat or intra-abdominal fat or ectopic fat or subcutaneous fat or overweight or obesity or adiposity or adipose or BMI or waist-to hip or waist-hip ratio or alcohol reduction or alcohol regulation or alcohol restriction:ti,ab,kw Publication Year from 2000 to 2014 (Word variations have been searched)

#6 (early origin or maternal or gestational diabetes or birth weight or intra-uterine or infant feeding or breast feeding or child growth) and (Cardiometabolic or cardiovascular):ti,ab,kw Publication Year from 2000 to 2014 (Word variations have been searched)

#7 (task shifting or task-shifting or self management or self-management) and (cardiometabolic or cardiovascular):ti,ab,kw Publication Year from 2000 to 2014 (Word variations have been searched)

#8 (change* or gain or loss or management or status or control) and weight:ti,ab,kw Publication Year from 2000 to 2014 (Word variations have been searched)

#9 #1 or #2 or #3 or #4 or #5 or #6 or #7 or #8

#10 developing countries or developing country or (low and middle income countries) or LMIC or south America or latin America or Afghanistan or Albania or Algeria or Angola or Argentina or Armenia or Armenian or Azerbaijan or Bangladesh or Benin or Belize or Bhutan or Bolivia or Botswana or Brazil or Burkina Faso or Burundi or Cambodia or Khmer Republic or Kampuchea or Cameroon or Cameroons or Cameron or Camerons or Cape Verde or Central African Republic or Chad or China or Colombia or Comoros or Comoro Islands or Comores or Mayotte or Congo or Zaire or Costa Rica or Cote d'Ivoire or Ivory Coast or Djibouti or French Somaliland or Dominica or Dominican Republic or East Timor or East Timur or Timor Leste or Ecuador or Egypt or United Arab Republic or El Salvador or Eritrea or Ethiopia or Fiji or Gabon or Gabonese Republic or Gambia or Gaza or Georgia or Ghana or Grenada or Guatemala or Guinea or Guiana or Guyana or Haiti or Honduras or India or Maldives or Indonesia or Kenya or Kiribati or Lao PDR or Laos or Lesotho or Basutoland or Liberia or Libya or Madagascar or Malagasy Republic or Sabah or Sarawak or Malawi or Nyasaland or Mali or Marshall Islands or Mauritania or Mauritius or Agalega Islands or Mexico or Micronesia or Moldova or Moldovia or Moldovian or Mongolia or Montenegro or Morocco or Ifni or Mozambique or Myanmar or Myanma or Burma or Namibia or Nepal or Netherlands Antilles or Nicaragua or Niger or Nigeria or Muscat or Pakistan or Palau or Palestine or Panama or Paraguay or Peru or Philippines or Philipines or Phillipines or Phillippines or Rwanda or Ruanda or Nevis or Saint Lucia or St Lucia or Saint Vincent or St Vincent or Grenadines or Samoa or Samoan Islands or Navigator Island or Navigator Islands or Sao Tome or Senegal or Serbia or Montenegro or Seychelles or Sierra Leone or Sri Lanka or Ceylon or Solomon Islands or Somalia or Sudan or Suriname or Surinam or Swaziland or Tajikistan or Tadzhikistan or Tadjikistan or Tadzhik or Tanzania or Thailand or Togo or Togolese Republic or Tonga or Tunisia or Turkey or Turkmenistan or Turkmen or Uganda or Ukraine or Vanuatu or New Hebrides or Venezuela or Vietnam or Viet Nam or Zambia or Zimbabwe or Africa, Northern or Northern Africa or North Africa or Africa South of the Sahara or sub-Saharan Africa or subsaharan Africa or Africa, Central or central Africa or Africa, Eastern or Eastern Africa or east Africa or Africa, Southern or southern Africa or Africa, Western or western Africa or west Africa or Caribbean Region or Caribbean or Central America or Panama Canal Zone or French Guiana or Borneo or Mekong Valley or mekong delta or Republic of Congo or Congo-Brazzaville or Democratic Republic of the Congo or DRC or Congo-Kishasha or South Sudan or South Africa or Guinea-Bissau:ti,ab,kw Publication Year from 2000 to 2014 (Word variations have been searched)

#11 #9 and #10

#12 cost or cost analysis or economics or cost savings or cost of illness or health care costs or direct service costs or drug costs or hospital costs or health expenditures or cost effectiveness or cost-effectiveness or cost of treatment or cost of disease or cost of care or health care cost or economic evaluation or cost analysis or economic analysis or cost benefit analysis or cost allocation or cost of services or medicine cost or hospital cost or out-of-pocket or expenses or expenditure or QALY or DALY:ti,ab,kw Publication Year from 2000 to 2014 (Word variations have been searched)

#13 #11 and #12

**ECONLIT**

| **Query** | **Limiters/Expanders** |
| --- | --- |
| S16 | S14 AND S15 |
| S15 | AB ( cost or cost analysis or economics or cost savings or cost of illness or health care costs or direct service costs or drug costs or hospital costs or health expenditures or cost effectiveness or cost-effectiveness or cost of treatment or cost of disease or cost of care or health care cost or economic evaluation or cost analysis or economic analysis or cost benefit analysis or cost allocation or cost of services or medicine cost or hospital cost or out-of-pocket or expenses or expenditure or QALY or DALY ) OR TI ( cost or cost analysis or economics or cost savings or cost of illness or health care costs or direct service costs or drug costs or hospital costs or health expenditures or cost effectiveness or cost-effectiveness or cost of treatment or cost of disease or cost of care or health care cost or economic evaluation or cost analysis or economic analysis or cost benefit analysis or cost allocation or cost of services or medicine cost or hospital cost or out-of-pocket or expenses or expenditure or QALY or DALY ) |
| S14 | S12 AND S13 |
| S13 | AB ( developing countries or developing country or (low and middle income countries) or LMIC or south America or latin America or Afghanistan or Albania or Algeria or Angola or Argentina or Armenia or Armenian or Azerbaijan or Bangladesh or Benin or Belize or Bhutan or Bolivia or Botswana or Brazil or Burkina Faso or Burundi or Cambodia or Khmer Republic or Kampuchea or Cameroon or Cameroons or Cameron or Camerons or Cape Verde or Central African Republic or Chad or China or Colombia or Comoros or Comoro Islands or Comores or Mayotte or Congo or Zaire or Costa Rica or Cote d'Ivoire or Ivory Coast or Djibouti or French Somaliland or Dominica or Dominican Republic or East Timor or East Timur or Timor Leste or Ecuador or Egypt or United Arab Republic or El Salvador or Eritrea or Ethiopia or Fiji or Gabon or Gabonese Republic or Gambia or Gaza or Georgia or Ghana or Grenada or Guatemala or Guinea or Guiana or Guyana or Haiti or Honduras or India or Maldives or Indonesia or Kenya or Kiribati or Lao PDR or Laos or Lesotho or Basutoland or Liberia or Libya or Madagascar or Malagasy Republic or Sabah or Sarawak or Malawi or Nyasaland or Mali or Marshall Islands or Mauritania or Mauritius or Agalega Islands or Mexico or Micronesia or Moldova or Moldovia or Moldovian or Mongolia or Montenegro or Morocco or Ifni or Mozambique or Myanmar or Myanma or Burma or Namibia or Nepal or Netherlands Antilles or Nicaragua or Niger or Nigeria or Muscat or Pakistan or Palau or Palestine or Panama or Paraguay or Peru or Philippines or Philipines or Phillipines or Phillippines or Rwanda or Ruanda or Nevis or Saint Lucia or St Lucia or Saint Vincent or St Vincent or Grenadines or Samoa or Samoan Islands or Navigator Island or Navigator Islands or Sao Tome or Senegal or Serbia or Montenegro or Seychelles or Sierra Leone or Sri Lanka or Ceylon or Solomon Islands or Somalia or Sudan or Suriname or Surinam or Swaziland or Tajikistan or Tadzhikistan or Tadjikistan or Tadzhik or Tanzania or Thailand or Togo or Togolese Republic or Tonga or Tunisia or Turkey or Turkmenistan or Turkmen or Uganda or Ukraine or Vanuatu or New Hebrides or Venezuela or Vietnam or Viet Nam or Zambia or Zimbabwe or Africa, Northern or Northern Africa or North Africa or Africa South of the Sahara or sub-Saharan Africa or subsaharan Africa or Africa, Central or central Africa or Africa, Eastern or Eastern Africa or east Africa or Africa, Southern or southern Africa or Africa, Western or western Africa or west Africa or Caribbean Region or Caribbean or Central America or Panama Canal Zone or French Guiana or Borneo or Mekong Valley or mekong delta or Republic of Congo or Congo-Brazzaville or Democratic Republic of the Congo or DRC or Congo-Kishasha or South Sudan or South Africa or Guinea-Bissau ) OR TI ( developing countries or developing country or (low and middle income countries) or LMIC or south America or latin America or Afghanistan or Albania or Algeria or Angola or Argentina or Armenia or Armenian or Azerbaijan or Bangladesh or Benin or Belize or Bhutan or Bolivia or Botswana or Brazil or Burkina Faso or Burundi or Cambodia or Khmer Republic or Kampuchea or Cameroon or Cameroons or Cameron or Camerons or Cape Verde or Central African Republic or Chad or China or Colombia or Comoros or Comoro Islands or Comores or Mayotte or Congo or Zaire or Costa Rica or Cote d'Ivoire or Ivory Coast or Djibouti or French Somaliland or Dominica or Dominican Republic or East Timor or East Timur or Timor Leste or Ecuador or Egypt or United Arab Republic or El Salvador or Eritrea or Ethiopia or Fiji or Gabon or Gabonese Republic or Gambia or Gaza or Georgia or Ghana or Grenada or Guatemala or Guinea or Guiana or Guyana or Haiti or Honduras or India or Maldives or Indonesia or Kenya or Kiribati or Lao PDR or Laos or Lesotho or Basutoland or Liberia or Libya or Madagascar or Malagasy Republic or Sabah or Sarawak or Malawi or Nyasaland or Mali or Marshall Islands or Mauritania or Mauritius or Agalega Islands or Mexico or Micronesia or Moldova or Moldovia or Moldovian or Mongolia or Montenegro or Morocco or Ifni or Mozambique or Myanmar or Myanma or Burma or Namibia or Nepal or Netherlands Antilles or Nicaragua or Niger or Nigeria or Muscat or Pakistan or Palau or Palestine or Panama or Paraguay or Peru or Philippines or Philipines or Phillipines or Phillippines or Rwanda or Ruanda or Nevis or Saint Lucia or St Lucia or Saint Vincent or St Vincent or Grenadines or Samoa or Samoan Islands or Navigator Island or Navigator Islands or Sao Tome or Senegal or Serbia or Montenegro or Seychelles or Sierra Leone or Sri Lanka or Ceylon or Solomon Islands or Somalia or Sudan or Suriname or Surinam or Swaziland or Tajikistan or Tadzhikistan or Tadjikistan or Tadzhik or Tanzania or Thailand or Togo or Togolese Republic or Tonga or Tunisia or Turkey or Turkmenistan or Turkmen or Uganda or Ukraine or Vanuatu or New Hebrides or Venezuela or Vietnam or Viet Nam or Zambia or Zimbabwe or Africa, Northern or Northern Africa or North Africa or Africa South of the Sahara or sub-Saharan Africa or subsaharan Africa or Africa, Central or central Africa or Africa, Eastern or Eastern Africa or east Africa or Africa, Southern or southern Africa or Africa, Western or western Africa or west Africa or Caribbean Region or Caribbean or Central America or Panama Canal Zone or French Guiana or Borneo or Mekong Valley or mekong delta or Republic of Congo or Congo-Brazzaville or Democratic Republic of the Congo or DRC or Congo-Kishasha or South Sudan or South Africa or Guinea-Bissau ) |
| S12 | S1 OR S2 OR S3 OR S4 OR S5 OR S6 OR S7 OR S10 OR S11 |
| S11 | TI ( change* or gain or loss or management or status or control ) AND TI weight |
| S10 | AB ( change* or gain or loss or management or status or control ) AND AB weight |
| S9 | TI ( task shifting or task-shifting or self management or self-management ) AND TI ( cardiovascular OR cardiometabolic ) |
| S8 | AB ( task shifting or task-shifting or self management or self-management ) AND AB ( cardiovascular OR cardiometabolic ) |
| S7 | TI ( early origin or maternal or gestational diabetes or birth weight or intra-uterine or infant feeding or breast feeding or child growth ) AND TI ( cardiovascular OR cardiometabolic ) |
| S6 | AB ( early origin or maternal or gestational diabetes or birth weight or intra-uterine or infant feeding or breast feeding or child growth ) AND AB ( cardiovascular OR cardiometabolic ) |
| S5 | AB ( physical education or (fruits and vegetables) or sugary beverage or body weight or body mass index or waist circumference or body fat or fat mass or abdominal fat or intra-abdominal fat or ectopic fat or subcutaneous fat or overweight or obesity or adiposity or adipose or BMI or waist-to hip or waist-hip ratio or alcohol reduction or alcohol regulation or alcohol restriction ) OR TI ( physical education or (fruits and vegetables) or sugary beverage or body weight or body mass index or waist circumference or body fat or fat mass or abdominal fat or intra-abdominal fat or ectopic fat or subcutaneous fat or overweight or obesity or adiposity or adipose or BMI or waist-to hip or waist-hip ratio or alcohol reduction or alcohol regulation or alcohol restriction ) |
| S4 | AB ( haemodyalisis or Retinopathy or Acute kidney injury or Renal disease or Dialysis or rheumatic fever or rheumatic heart disease or congenital heart disease or myocarditis or valvular heart disease or infective endocarditis or low salt or salt free or low sodium or sodium restricted or salt restricted or sodium free or salt reduction or smoking cessation or tobacco cessation or smoking ban or tobacco control or physical activity or exercise promotion or health promotion or dietary improvement or vegetable consumption or fruit consumption or obesity reduction or unhealthy diet or healthy diet or impaired glucose tolerance ) OR TI ( haemodyalisis or Retinopathy or Acute kidney injury or Renal disease or Dialysis or rheumatic fever or rheumatic heart disease or congenital heart disease or myocarditis or valvular heart disease or infective endocarditis or low salt or salt free or low sodium or sodium restricted or salt restricted or sodium free or salt reduction or smoking cessation or tobacco cessation or smoking ban or tobacco control or physical activity or exercise promotion or health promotion or dietary improvement or vegetable consumption or fruit consumption or obesity reduction or unhealthy diet or healthy diet or impaired glucose tolerance ) |
| S3 | AB ( ejection fraction or systolic failure or stroke or aneurysm or atherosclerosis or cerebrovascular disease or peripheral artery disease or peripheral vascular disease or diabetes or diabetic foot or blood sugar or Type 2 DM or T2DM or insulin resistance or impaired glucose tolerance or impaired fasting glycaemia or hyperlipidaemia or hyperlipidemia or dyslipidemia or chronic respiratory disease or COPD or Chronic obstructive pulmonary disease or chronic airway obstruction or asthma or restrictive lung disease or lung fibrosis or lung scarring or chronic bronchitis or bronchitis or kidney disease or hemodialysis or end stage renal disease or ESRD or renal transplant or kidney transplant ) OR TI ( ejection fraction or systolic failure or stroke or aneurysm or atherosclerosis or cerebrovascular disease or peripheral artery disease or peripheral vascular disease or diabetes or diabetic foot or blood sugar or Type 2 DM or T2DM or insulin resistance or impaired glucose tolerance or impaired fasting glycaemia or hyperlipidaemia or hyperlipidemia or dyslipidemia or chronic respiratory disease or COPD or Chronic obstructive pulmonary disease or chronic airway obstruction or asthma or restrictive lung disease or lung fibrosis or lung scarring or chronic bronchitis or bronchitis or kidney disease or hemodialysis or end stage renal disease or ESRD or renal transplant or kidney transplant ) |
| S2 | AB ( stent or coronary heart disease or coronary syndrome or heart failure or hypertensive or anti-hypertension or cholesterol or ischemia or atherosclerosis or hypertension or high blood pressure or blood pressure control or angiography or ischemic heart disease or acute myocardial infarction or coronary bypass surgery or cardiomyopathies or non-communicable disease or noncommunicable disease or NCDs or ventricular dysfunction or Angina or Acute coronary syndrome or cardiac output ) OR TI ( stent or coronary heart disease or coronary syndrome or heart failure or hypertensive or anti-hypertension or cholesterol or ischemia or atherosclerosis or hypertension or high blood pressure or blood pressure control or angiography or ischemic heart disease or acute myocardial infarction or coronary bypass surgery or cardiomyopathies or non-communicable disease or noncommunicable disease or NCDs or ventricular dysfunction or Angina or Acute coronary syndrome or cardiac output ) |
| S1 | AB ( CVD or secondary prevention or heart disease or cardiovascular disease or cardiometabolic disease or cardiometabolic disorder or cardio-metabolic disease or cardio-metabolic disorder or CABG).ab. or (CVD or secondary prevention or heart disease or cardiovascular disease or cardiometabolic disease or cardiometabolic disorder or cardio-metabolic disease or cardio-metabolic disorder or CABG ) OR TI ( CVD or secondary prevention or heart disease or cardiovascular disease or cardiometabolic disease or cardiometabolic disorder or cardio-metabolic disease or cardio-metabolic disorder or CABG).ab. or (CVD or secondary prevention or heart disease or cardiovascular disease or cardiometabolic disease or cardiometabolic disorder or cardio-metabolic disease or cardio-metabolic disorder or CABG ) |

**HEED database =**

Cardiovascular OR overweight OR obesity OR hypertension OR angina OR diabetes OR stroke OR stent OR coronary OR ischemic OR diet OR fruit OR vegetable OR tobacco OR heart OR lung OR CABG OR CVD

AND

Developing countries OR LMIC OR low and middle income countries OR asia OR Africa OR latin America
